# Characterisation of bacteria representing a novel *Nitrosomonas* clade: Physiology, genomics and distribution of missing ammonia oxidizer

**DOI:** 10.1111/1758-2229.13158

**Published:** 2023-04-20

**Authors:** Shuta Kikuchi, Hirotsugu Fujitani, Kento Ishii, Rino Isshiki, Yuji Sekiguchi, Satoshi Tsuneda

**Affiliations:** ^1^ Department of Life Science and Medical Bioscience Waseda University Tokyo Japan; ^2^ Department of Biological Sciences Chuo University Tokyo Japan; ^3^ Research Organization for Nano & Life Innovation Waseda University Tokyo Japan; ^4^ Biomedical Research Institute National Institute of Advanced Industrial Science and Technology (AIST) Ibaraki Japan

## Abstract

Members of the genus *Nitrosomonas* are major ammonia oxidizers that catalyse the first step of nitrification in various ecosystems. To date, six subgenus‐level clades have been identified. We have previously isolated novel ammonia oxidizers from an additional clade (unclassified cluster 1) of the genus *Nitrosomonas*. In this study, we report unique physiological and genomic properties of the strain PY1, compared with representative ammonia‐oxidising bacteria (AOB). The apparent half‐saturation constant for total ammonia nitrogen and maximum velocity of strain PY1 were 57.9 ± 4.8 μM NH_3_ + NH_4_^+^ and 18.5 ± 1.8 μmol N (mg protein)^−1^ h^−1^, respectively. Phylogenetic analysis based on genomic information revealed that strain PY1 belongs to a novel clade of the *Nitrosomonas* genus. Although PY1 contained genes to withstand oxidative stress, cell growth of PY1 required catalase to scavenge hydrogen peroxide. Environmental distribution analysis revealed that the novel clade containing PY1‐like sequences is predominant in oligotrophic freshwater. Taken together, the strain PY1 had a longer generation time, higher yield and required reactive oxygen species (ROS) scavengers to oxidize ammonia, compared with known AOB. These findings expand our knowledge of the ecophysiology and genomic diversity of ammonia‐oxidising *Nitrosomonas*.

## INTRODUCTION

Nitrification, the microbial oxidation of ammonia to nitrate via nitrite, is crucial for biogeochemical nitrogen cycling and artificial nitrogen removal. Nitrification is performed by ammonia‐oxidising bacteria (AOB), ammonia‐oxidising archaea (AOA), nitrite‐oxidising bacteria (NOB) or a single microorganism (complete ammonia oxidizer [comammox]) (Daims et al., 2015; van Kessel et al., 2015). The first AOB, *Nitrosomonas europaea*, was reported over 130 years ago (Winogradsky, 1890). Since then, many AOB have been detected in diverse environments and engineered ecosystems (Purkhold et al., 2000). AOB phylogeny is categorised into two monophyletic orders within Gammaproteobacteria: Betaproteobacteriales and Nitrosococcales. AOB within Betaproteobacteriales are divided into the genera *Nitrosomonas* and *Nitrosospira*. *Nitrosomonas* AOB comprises six clusters (5, 6a, 6b, 7, 8 and 9) based on either the 16S rRNA or *amoA* gene phylogeny (Koops et al., 2006). Based on 16S rRNA or *amoA* genes, the phylogeny of *Nitrosomonas* AOB is correlated with physiological adaptation (Kowalchuk & Stephen, 2001; Stephen et al., 1996). For instance, cluster 6a AOB (*Nitrosomonas oligotropha* lineage) favour oligotrophic environments with high ammonia affinity, whereas cluster 7 AOB (*N. europaea/mobilis* lineage) are adapted to eutrophic environments with low ammonia affinity (Koops et al., 2006; Koops & Pommerening‐Röser, 2001). Recent comparative genomic data support these physiological traits and highlight the differences between clusters 6a and 7 AOB (Sedlacek et al., 2019). Cluster‐specific physiological, genomic and species‐specific adaptations have helped explain the differential responses of AOB communities to environmental changes (Sedlacek et al., 2019).

Data concerning the physiology, genome, phylogeny and distribution of AOB have accumulated steadily over the last 130 years. Understanding AOB has considerably depended on the study of axenic cultures (Stein, 2019). However, AOB gain ATP via the uptake of ammonia as the sole energy source. The consequent production of nitrite and decreased pH cause a decrease in AOB activity (Claros et al., 2013). Once AOB are isolated as pure cultures, they are exposed to elevated nitrite concentrations and acidification. This inevitable condition often impedes long‐term preservation and recovery and can result in the loss of AOB isolates (Bollmann et al., 2011; Fujitani et al., 2015). AOB, which are fastidious and recalcitrant microorganisms in the laboratory setting and have not been cultured and isolated, have attracted much research attention. Recent culture‐based studies have reported a new phylogeny of AOB adapted to acidic conditions (Hayatsu et al., 2017; Picone et al., 2021), indicating that the environmental diversity of AOB remains unclear.

We previously reported the isolation of novel AOB retrieved from activated sludge, which were affiliated with an additional clade (unclassified cluster 1) of the *Nitrosomonas* genus at the 16S rRNA and *amoA* gene levels (Abe et al., 2017). The isolates were obtained by detecting clonal microcolonies in activated sludge using fluorescence in situ hybridization and sorting microcolonies based on scattering signatures. However, the ecophysiology and genomics of these isolates remain unclear. In this study, we report the physiology and genome of one of the isolates, which was categorised as a previously unrecognised clade of the *Nitrosomonas* genus. The abundance and environmental distribution of the new *Nitrosomonas* clade was investigated using an integrated microbial next‐generation sequencing platform (IMNGS).

## RESULTS AND DISCUSSION

### Effect of catalase on the growth of strain PY1

After isolating and identifying three AOB (strains SN1, NP1 and PY1) retrieved from activated sludge (Abe et al., 2017), we established a protocol to obtain sufficient biomass for physiological experiments and genome sequencing. The three strains were cultivated and transferred to an inorganic medium containing ammonium for 2 years. Two strains, SN1 and NP1, were lost and the ammonia oxidation activity of PY1 remained low. Even if 0.56 mM NH_4_Cl was added to the culture, only 0.1 mM nitrite was produced (Figure S1). To overcome this drawback, we added various substances, including carbohydrates, organic acids, amino acids, complex nutrients and urea, to inorganic media to enhance the ammonia‐oxidising activity of PY1; however, these attempts failed (Table S1). In a previous study, adding a hydrogen peroxide (H_2_O_2_) scavenger stimulated the ammonia oxidation of *Nitrosopumilus* sp. DDS1 (Kim et al., 2016). Based on this knowledge, we cultivated PY1 in ammonium‐based medium supplemented with catalase at 50 or 400 U mL^−1^. Consequently, PY1 showed improved ammonia oxidation activity and produced more nitrite (Figure S1 and Table S1). Unless otherwise stated, inorganic media containing catalase were utilised for strain PY1 in subsequent experiments.

### Physiological characteristics

**FIGURE 1:**
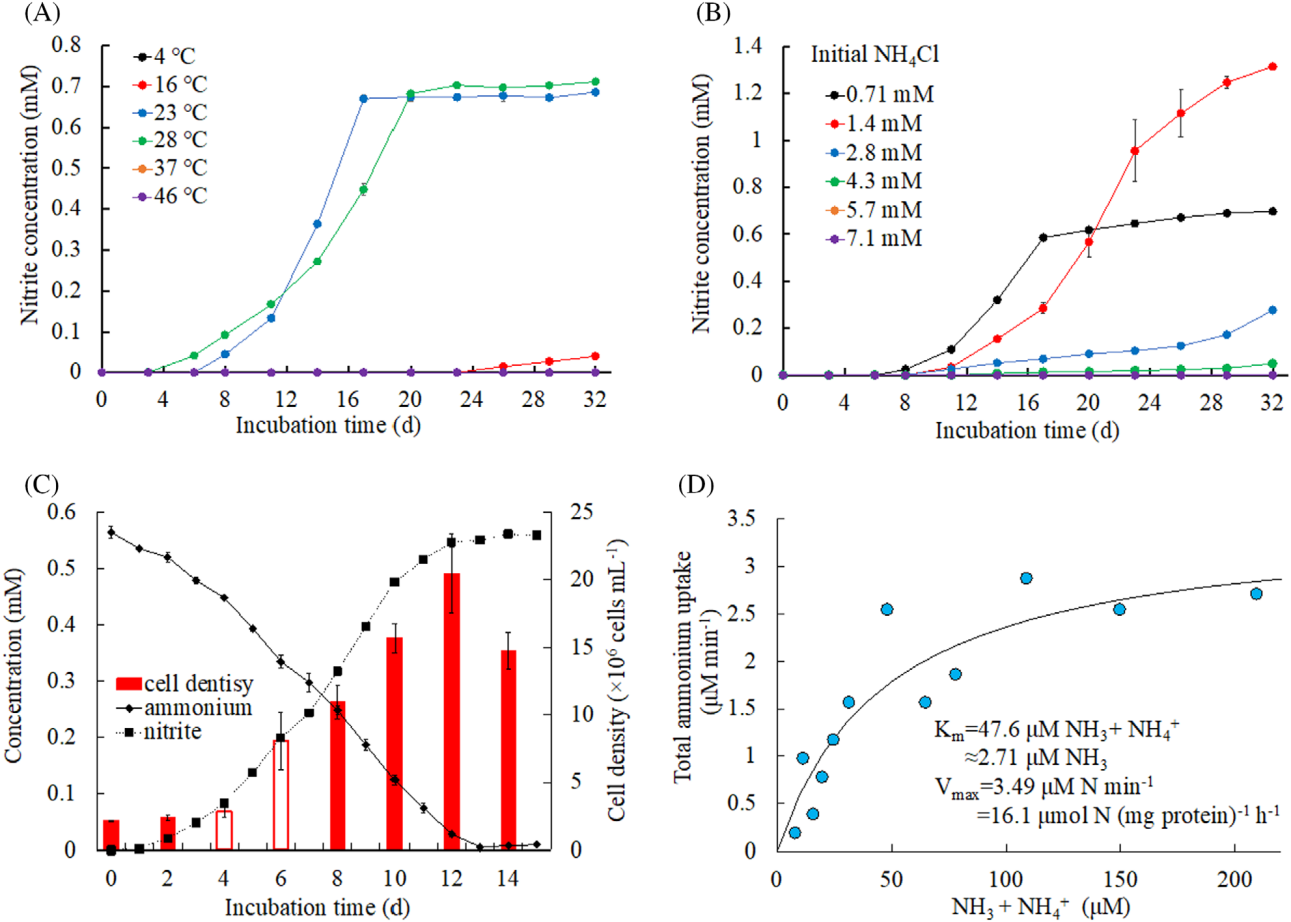
Physiological characteristics of strain PY1. (A) Temperature dependence of nitrite production. The black, orange and purple circles completely overlapped because nitrite production was not observed at 4°C, 37°C and 46°C. (B) Influence of initial ammonium concentration on ammonia oxidation activity. The orange and purple circles completely overlapped because nitrite production was not observed at initial NH_4_Cl concentrations of 5.7 mM and 7.1 mM. (C) Changes in ammonium and nitrite concentrations as well as cell density estimated by the 16S rRNA gene copies of Betaproteobacterial AOB using the qPCR analysis. White bars on days 4 and 6 were used to calculate the maximum specific growth rate. (D) Representative data showing the ammonia uptake activity. The curve was fitted to the Michaelis–Menten equation: Error bars depicting the standard deviations of triplicate biological measurements are shown; they are not shown if the bars are smaller than the symbols.

Physiological experiments were conducted to determine the optimum temperature (Figure 1A), ammonium concentration (Figure 1B), growth rate (Figure 1C) and kinetics (Figure 1D). Strain PY1 was cultivated at temperatures ranging from 4°C to 46°C, and nitrite was produced only at temperatures ranging from 23°C to 28°C with a total consumption of 0.71 mM ammonium for 2–3 weeks (Figure 1A). Compared to other ammonia oxidizers, including *Nitrosomonas* species, strain PY1 required a relatively narrow temperature range (23°C–28°C) to oxidize ammonia. However, the optimal temperature for the PY1 strain was typical of ammonia oxidizers (Table 1).

**TABLE 1:** Physiologic characteristics of Nitrosomonas sp. PY1 and other selected members of AOB.

Organism	Cluster	Generation time* (d)	Half‐saturation constant K_m(app)_ ^†^ (μM NH_3_)	Maximum total ammonium uptake rate V_max_ (μmol N mg protein^−1^ h^−1^)	Yield (cells pmol^−1^)	Maximum ammonium tolerance^‡^ (mM)	Optimum temperature (°C)	Origin	Reference
*Nitrosomonas* sp. PY1	PY1 cluster	3	2.7	18.5 ± 1.8	33.5	5.7	23–28	Activated sludge	This study
*Nitrsosomonas* sp. Is79A3	Cluster 6a	1.3	3.3, 3.6	ND	ND	ND	ND	Freshwater sediment	Sedlacek et al., 2016
*Nitrsosomonas* sp. AL212	Cluster 6a	ND	0.49^a^	ND	ND	71.4^b^	ND	Activated sludge	Suwa et al., 1994,^b^ Koper et al., 2010 ^a^
*Nitrosomonas oligtoropha*	Cluster 6a	ND	4.4^c^	ND	ND	50^d^	ND	Soil	Koops et al., 1991,^d^ Stehr et al., 1995 ^c^
*Nitrosomonas mobilis* Ms1	Cluster 7	0.49^§^	2.4	ND	0.489	200	27	Nitrifying granules	Thandar et al., 2016
*Nitrosomonas europaea*	Cluster 7	0.48–0.56^e^	23^f^	36^g^, 122^h^	2.04^i^ ^#^, 14.5^j^	400^d^	25‐30^k^	Wastewater, soil	Watson et al., 1971,^k^ Suzuki et al., 1974,^f^ Belser & Schmidt, 1980,^e^ Koops et al., 1991,^d^ Laanbroek & Gerards, 1993,^j^ Martens‐Habbena et al., 2009,^g^ Park et al., 2010,^h^ Kits et al., 2017 ^i^
*Nitrosomonas cryotolerans*	*N. cryotolerans* linage	ND	7.2^l^, 42‐59^m^	ND	ND	400^d^	5‐30^l^	Seawater	Jones & Morita, 1985,^l^ Koops et al., 1991,^d^ Koops & Pommerening‐Röser, 2001 ^m^
*Nitrosospira lacus* APG3	Cluster 0	ND	ND	ND	ND	200	20–25	Sandy lake sediment	Urakawa et al., 2015
*Nitrosospira briensis*	Cluster 3	ND	2.9^n^	ND	ND	200°	ND	Soil	Bollmann et al., 2005,^n^ Koops et al., 2006°
*Nitrosospira* sp. AF	Cluster 3	5.8	6	ND	0.7	ND	ND	Acid soil	Jiang & Bakken, 1999
*Nitrosococcus oceani*	‐	2.1^p^	15.6^q^	38.4^g^	0.378^i^ ^#^	1000^d^	ND	Seawater	Glover, 1985,^p^ Ward, 1987,^q^ Koops et al., 1991,^d^ Martens‐Habbena et al., 2009,^g^ Kits et al., 2017 ^i^
Ca. Nitrosoglobus terrae TAO100	‐	1.1	33.3	ND	ND	ND	25	Acid soil	Hayatsu et al., 2017
Ca. Nitrosacidococcus tergens RJ19	‐	ND	0.147	ND	ND	ND	ND	Biofilm unit of a pig farm	Picone et al., 2021

*Note*: When the generation time was not described, they were calculated using the following fomula: generation time = Ln(2)/μ, μ: specific growth rate. When the unit of generation time were hours (h), it was divided by 24 and converted to days (d). Superscripted alphabets show values determined in previous studies.

Abbreviation: ND, not determined.

* The generation time were applied from the values estimated from the exponential growth curve.

^†^ Km(app) (μM NH3) values were calculated by the formula described in Anthonisen et al. (1976), when Km(app) (μM NH3 + NH4+) values were only indicated in the previous study.

^‡^ The maximum ammonium tolerance concentration was considered to be the minimum initial ammonium concentration in the absence of nitrite production or bacterial growth.

^§^ This value was the minimum generation time that was calculated by maximum specific growth rate.

^#^ The yields were converted using cell biomass factors (120 and 650 fg protein cell−1 of *N. europaea* and *N. oceani*, respectively). (Martens‐Habbena et al., 2009).

To investigate tolerance to ammonium concentrations, PY1 was cultivated with different initial ammonium concentrations. During the 32 days of incubation, 0.71 mM and 1.4 mM ammonium were completely consumed, and equivalent nitrite was produced (Figure 1B). However, an initial ammonium concentration exceeding 2.8 mM delayed nitrite production, resulting in prolonged lag time. An initial ammonium concentration of 5.7 or 7.1 mM completely prevented nitrite production by the strain PY1. The maximum ammonium tolerance of PY1 was considerably lower than that reported for other AOB (Table 1).

The growth of PY1 during ammonia oxidation was evaluated using qPCR with 16S rRNA gene primers. After 14 days of incubation, cell density increased with the stoichiometric oxidation of ammonia to nitrite (Figure 1C). The specific growth rate calculated by fitting the cell growth curve to an exponential equation, was 0.23 day^−1^ (Figure S2). The maximum specific growth rate was 0.49 ± 0.20 day^−1^. The generation time was 3.0 days, which was longer than that of most ammonia oxidizers, except for *Nitrosospira* sp. AF isolated from acidic soil (Table 1). The estimated growth yield of PY1 was estimated to be 33.5 cells pmol^−1^.

The apparent half‐saturation constant (*K*_m (app)_) for total ammonia nitrogen (NH_3_ and NH_4_^+^) and maximum velocity (*V*_max_) were calculated by manually fitting the data obtained to the Michaelis–Menten equation (Figure 1D, Figure S3). Based on experiments performed in three biological replicates, the *K*_m (app)_ and *V*_max_ of strain PY1 were 57.9 ± 4.8 μM NH_3_ + NH_4_^+^ (3.3 μM NH_3_) and 18.5 ± 1.8 μmol N (mg protein)^−1^ h^−1^, respectively. The *K*_m (app)_ of strain PY1 was within the range of *Nitrosomonas* cluster 6a AOB, whereas the *V*_max_ of strain PY1 was lower than that of other AOB (Table 1). Considering these physiological properties, PY1 appears to be a typical AOB adapted to oligotrophic environments.

### Morphology

**FIGURE 2:**
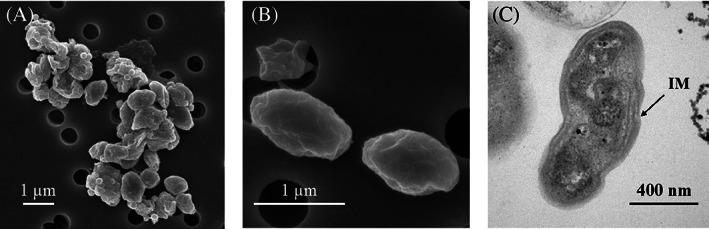
Electron micrographs of strain PY1. (A) Cell aggregation and (B) individual cells observed by scanning electron microscopy. (C) Ultrathin section of a cell observed using transmission electron microscopy. IM, intracytoplasmic membranes.

Scanning electron microscopy (SEM) revealed that PY1 cells were rod‐shaped, similar to previously isolated strains (Koops et al., 1991). As strain PY1 was isolated by sorting pure microcolonies from activated sludge samples (Abe et al., 2017), this strain may form cell aggregates (Figure 2A). The width and length of the rod‐shaped cells ranged from 0.9 to 1.6 μm and 0.5 to 0.8 μm, respectively (Figure 2B). Ultrathin sections of the cells were observed using transmission electron microscopy (TEM). As observed in other strains of the genus *Nitrosomonas* (Koops et al., 2006), PY1 had multilayer intracytoplasmic membranes (Figure 2C).

### Genome overview

**FIGURE 3:**
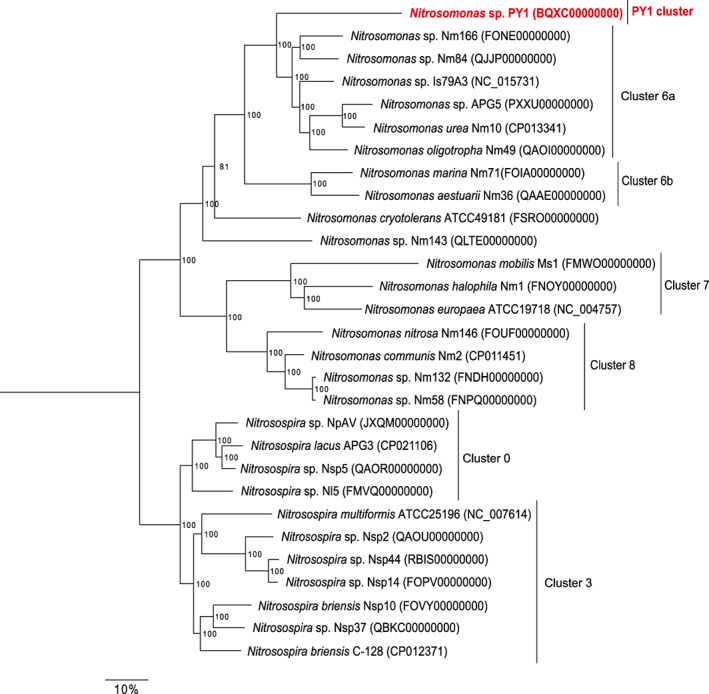
The genome tree based on the amino acid sequences of 120 single‐copy marker genes shared between representative *Nitrosomonas* and *Nitrosospira* genomes using the randomised accelerated maximum likelihood (RAxML) software (the JTT and the Gamma models with rapid 100‐times bootstrapping). Numbers at branch nodes indicate all bootstrap values (%).

The high‐quality draft genome of PY1, comprising four scaffolds, was reconstructed using paired‐end sequences and a mate‐pair sequence. The four scaffold sizes were 2,710,791 bp (scaffold 1), 58,489 bp (scaffold 2), 58,786 bp (scaffold 3) and 26,708 bp (scaffold 4). The total size of the genome was 2,854,774 bp. The G + C content was 42.7%. The genome contained 2612 predicted protein‐coding DNA sequences (CDS), a coding ratio of 86.6%, two rRNA genes (16S and 23S), 37 tRNA genes and one CRISPR gene (Table S2). The range of the average nucleotide identity (ANI) between strain PY1 and other AOB isolates was 76.7%–82.9% (Table 2). Considering that the species cut‐off point for ANI is 95% (Konstantinidis & Tiedje, 2005), strain PY1 was considered a novel AOB, at least at the species level. The range of the average amino acid identity (AAI) between strain PY1 and other AOB isolates was 60.2%–70.8% (Table 2). In addition, a genome tree revealed that PY1 is phylogenetically separated from the well‐known AOB cluster (Figure 3). Previously, we reported that the identity between strain PY1 and its closest species was 95% and 88% for 16S rRNA and *amoA* genes, respectively, and categorised the phylogeny of strain PY1 into a novel clade within the genus *Nitrosomonas* (Abe et al., 2017, Figure S4). This assignment was validated using the results of the genome tree constructed in this study.

**TABLE 2:** Genome features of Nitrosomonas sp. PY1 and some AOB species.

	Genome features	*Nitrosomonas* sp. PY1	*Nitrosomonas* sp. Is79A3	*Nitrosomonas* sp. AL212	*Nitrosomonas ureae* Nm10	*Nitrosomonas cryotolerans* ATCC 49181	*Nitrosomonas communis* Nm2	*Nitrosomonas mobilis* Ms1	*Nitrosomonas eutropha* C91	*Nitrosomonas europaea ATCC 19718*	*Nitrosospira multiformis* ATCC 25196
	Cluster	PY1 cluster	Cluster 6a	Cluster 6a	Cluster 6a	*N. cryotolerans* cluster	Cluster 8	Cluster 7	Cluster 7	Cluster 7	Cluster 3
	ANI (%)^†^	†	76.7	82.1	78.4	77.2	78.8	82.9	78.2	80.0	81.3
	AAI (%)^†^	†	70.8	70.6	70.5	65.9	62.8	60.2	61.0	60.9	60.9
Nitrogen metabolism	Ammonia monooxygenase (amoCAB)	3	3	3	3	3	2	2	2	2	3
	ORF4(amoE)	3	2	3	3	2	2	2	2	2	2
	ORF5(amoD)	2	2	2	2	2	2	1	2	2	2
	Copper transport and resistance proteins (copCD)	1	0	1	1	1	1	N/A	1	1	2
	Singleton amoC	2	2	2	2	2	0	0	1	1	2
	Hydroxylamine oxidoreductase (haoAB)	3	3	3	4	3	2	2	3	3	3
	Cytochrome *c*‐554 (cycA)	3	3	3	4	3	2	2	3	3	3
	Cytochrome C_M_‐554 (cycB)	3	3	3	4	3	1	2	2	2	3
	Nitrosocyanin (NcyA)	+	−	+	+	+	+	+	+	+	+
	Copper‐containing nitrite reductase (NirK)	+	+	+	+	+	−	+	+	+	+
	Nitric oxide reductase cNOR (norCBQD)	+	−	+	−	+	+	+	+	+	+
	Haem‐copper nitric oxide reductase sNOR (norS‐SenC)	−	−	−	−	−	+	+	+	+	+
	Cytochrome *c*’ beta (cytS)	1	1	1	1	N/A	1	1	1	1	1
	Cytochrome P460 (cytL)	2	1	2	1	2	2	2	1	1	0
	NO‐responsive transcriptional regulator (NsrR)	0	0	0	0	N/A	0	N/A	1	1	0
	NO‐responsive transcriptional regulator (NnrR)	1	1	1	1	N/A	2	N/A	1	1	0
	Ammonia transporter	−	+	+	+	−	−	+	−	+	+
	Urease	+	−	+	+	+	−	−	−	−	+
Carbon fixation	RuBisCO	1	2	2	2	1	2	1	1	1	1
		(form IA)	(form IA, IC)	(form IA, IC)	(form IA, IC)	(form IA)	(form IA, IC)	(form IA)	(form IA)	(form IA)	(form IC)
Oxidative stress	Superoxide dismutase	1	1	1	1	1	1	1	1	1	1
	Catalase	1	1	2	2	1	2	1	1	2	1
	Rubrerythrin (Rbx)	1	1	1	1	1	−	1	−	−	1
	Alkyl hydroperoxide reductase (AhpC)	+	+	+	+	+	+	+	+	+	+
	Transaldolase (tal)	−	+	−	−	+	+	+	+	+	+
	Origin	Activated sludge	Freshwater sediment	Activated sludge	Soil	Seawater	Soil	Nitrifying granules	Municipal sewage	Wastewater	Soil
	GenBank sequence	−	Nit79A3_2491	CP002552.1‐CP002554.1	CP013341.1	FSRO01000001.1‐FSRO01000002.1	CP011451.1	FMWO01000001.1‐ FMWO01000112.1	CP000450.1‐CP000452.1	AL954747.1	CP000103.1‐CP000106.1
	Reference	This study	Bollmann et al., 2013	Suwa et al., 2011	Kozlowski et al., 2016a, Kozlowski et al., 2016c	Rice et al., 2017	Kozlowski et al., 2016b, Kozlowski et al., 2016c	Thandar et al., 2016	Stein et al., 2007	Chain et al., 2003	Norton et al., 2008

Abbreviations: +, positive; −, negative; N/A, not applicable.

^†^ Average nucleotide identity (ANI) and average amino acids identity (AAI) of PY1 strain with other AOB pure strains.

### Nitrogen metabolism

**FIGURE 4:**
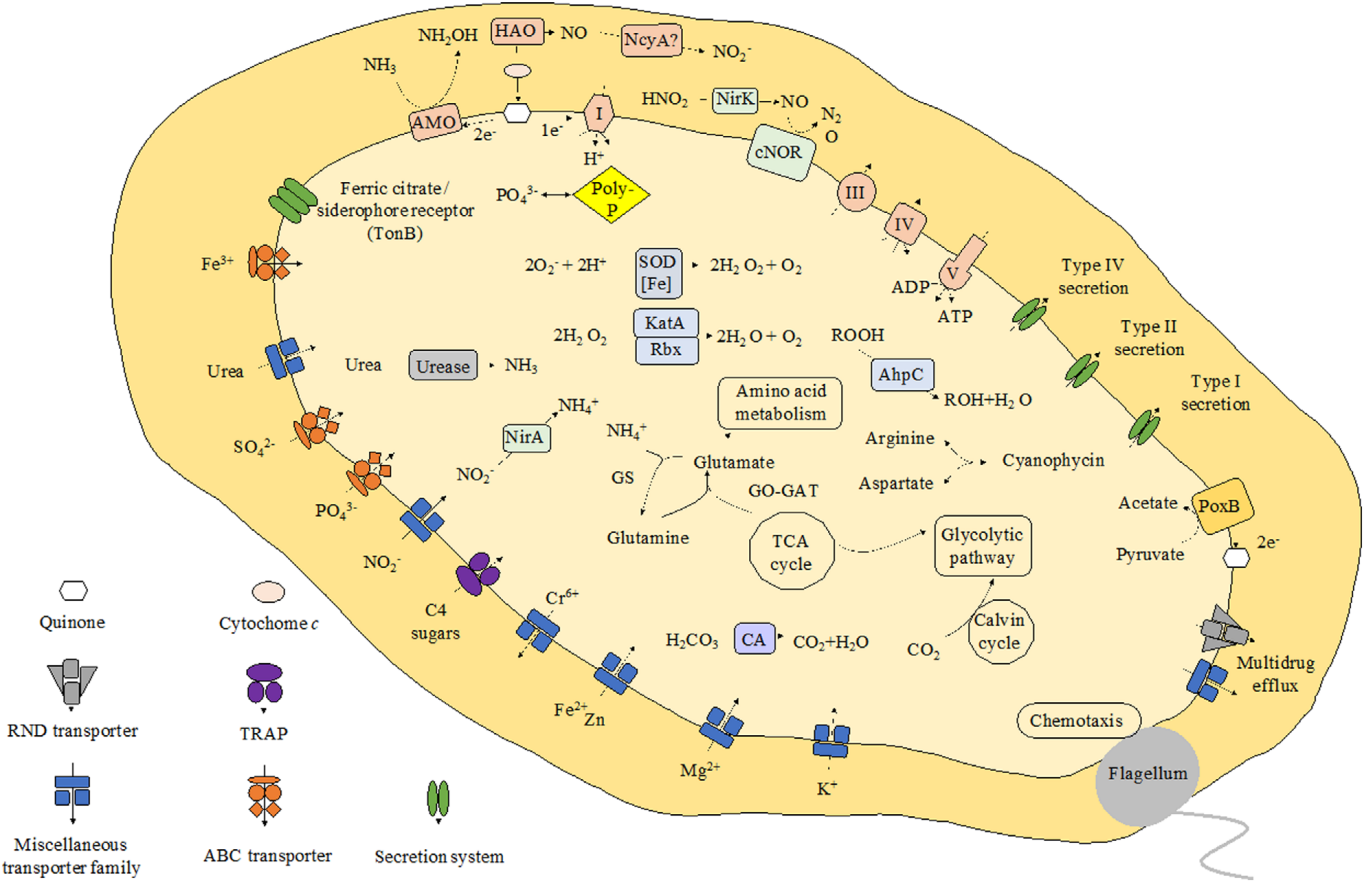
Metabolic cartoon of strain PY1 based on the genes annotated using the DDBJ Fast Annotation and Submission Tool (DFAST). AhpC, Alkyl hydroperoxide reductase; AMO, ammonia monooxygenase; CA, carbonic anhydrase; cNOR, nitric oxide reductase; HAO, hydroxylamine dehydrogenase; KatA, catalase; NcyA, nitrocyanin; NirA, nitrite reductase; NirK, copper‐containing nitrite reductase; PoxB, pyruvate dehydrogenase (ubiquinone); Rbx, rubrerythrin; SOD [Fe], Superoxide dismutase [Fe].

The PY1 genome contained three copies of the gene cluster encoding ammonia monooxygenase (*amoCAB*), followed by two copies of the *amoED* operon. The genes likely encoding the copper transport and residence proteins *copC* and *copD* were detected downstream of one of these *amoCABED* operons. The genome of strain PY1 contains two copies of the singleton *amoC* followed by one copy of *amoE*. PY1 potentially encodes three complete operons for hydroxylamine dehydrogenase (*haoAB*‐*cysAB*), copper‐containing nitrite reductase (*nirK*), nitric oxide reductase (*norCBQD*) and nitrosocyanin (*ncyA*) (Figure 4, Table S3). Recently, *nirK* and *ncyA* have been suggested as candidates for nitric oxide oxidoreductase (NOO), a newly proposed enzyme in the pathway for energy conservation by AOB (Caranto & Lancaster, 2017; Zorz et al., 2018). The copy numbers of the *amoCAB*, singleton *amoC* and *haoAB*‐*cysAB* operons were almost the same as those of cluster 6a AOB (*Nitrosomonas* sp. Is79A3 and *Nitrosomonas* sp. AL212, *N. ureae*) (Table 2).

Although the genomes of some AOB contain an ammonia transporter, none were detected in the PY1 genome, which is similar to the *N. eutropha* C91 genome (Stein et al., 2007) (Table 2). Strain PY1 and other AOB (Hayatsu et al., 2017; Offre et al., 2014) may incorporate ammonia via passive diffusion. These properties could be disadvantageous for PY1, hindering its adaptation to environments with variable ammonium/ammonia concentrations. Furthermore, strain PY1 was more sensitive to higher ammonium concentrations than the other AOB (Table 1). The lack of control of ammonium concentration within the cytoplasm might be responsible for the difficulty in cultivating PY1 in a laboratory setting. Additionally, PY1 has a potential formate/nitrite transporter (*focA*), which transfers nitrite to the cytoplasm and ferredoxin‐nitrite reductase (*nirA*), which reduces nitrite to ammonia (Figure 4). Strain PY1 contains a urea transporter that transfers urea to the cytoplasm, urease (*ureCBA*) and urease accessory protein (*ureDEFG*) (Koper et al., 2004) (Figure 4, Table S5). However, the addition of urea to the inorganic medium did not affect the growth of PY1 in the absence of catalase (Table S1). To determine whether strain PY1 can use urea as an energy source, we conducted an experiment on urea utilisation by PY1. In the presence of catalase, we found that urea was hydrolysed to ammonium, which was converted into nitrite. In the absence of catalase, urea was hydrolysed to ammonium, which accumulated without nitrite production (Figure S5). Therefore, PY1 can only use urea as an energy source in the presence of reactive oxygen species (ROS) scavengers.

In many AOB, the two‐electron reduction in two molecules of NO to N_2_O is performed by two classes of cytochrome *c* NOR (cNOR), encoded by the norCBQD operon, and the alternative NO reductase (sNOR) encoded by the norSY‐SenC operon (Kits et al., 2019). The PY1 genome contained only one copy of the gene for cNOR but no sNOR gene, similar to *Nitrosomonas* sp. AL212 and *N. cryotolerans* (Rice et al., 2017; Suwa et al., 2011). AOB species within clusters 7 and 8, which are adapted to eutrophic environments, tended to have two types of NOR. In contrast, AOB species within cluster 6a and the PY1 cluster, which are adapted to oligotrophic environments, had only cNOR or neither NOR. Therefore, the NOR setup might be specific to each AOB cluster. Considering that ammonia concentration is considerably low in oligotrophic environments where does not accumulate n‐oxides such as nitric oxide and hydroxylamine, NOR is not always required for oligotrophic AOB (Kozlowski et al., 2016c). However, nitric oxide and hydroxylamine frequently accumulate in wastewater treatment plants (WWTPs) and in laboratory cultures of activated sludge (Kampschreur et al., 2008; Wang et al., 2016; Yang & Alleman, 1992). Considering that PY1 and AL212 were isolated from activated sludge, it might be essential to have multiple mechanisms for oxidising the intermediate nitrogen metabolites of these species. In addition, PY1 harbours two copies of cytochrome P460 gene (*cytL*). CytL has been shown to oxidise nitric oxide and hydroxylamine to nitrite (Stein, 2011) and/or to oxidise hydroxylamine to nitrous oxide (Caranto et al., 2016). More recently, *Nitrosomonas*. sp AL212 CytL was biochemically characterised (Smith et al., 2019; Smith & Lancaster, 2018). Considering that the expression of CytL was confirmed during aerobic growth in some AOB, but not all tested AOB (Zorz et al., 2018), it remains unknown whether CytL in strain PY1 is functional.

### Carbon fixation

AOB potentially possess two types of ribulose‐1,5‐bisphosphate carboxylase/oxygenase (RuBisCO), type IA and type IC. The affinity for carbon dioxide is known to be higher for type IA than type IC (Badger & Bek, 2008). We observed that strain PY1 formed contained type IA RuBisCO, three copies of carbonic anhydrase and no carboxysome genes (Figure 4, Table S3). AOB affiliated with cluster 6a have one copy of each type of RuBisCO and thus a more flexible ability to fix carbon dioxide (Bollmann et al., 2013). In contrast, strain PY1 has only type IA RuBisCO. Similar to strain PY1, cluster 7 AOB possesses only the type IA RuBisCO, whereas some but not all cluster 7 AOB encode carboxysome genes (Sedlacek et al., 2019). Therefore, there seems to be no correlation between RuBisCO and carboxysome genes. Strain PY1 might require a more stringent carbon dioxide concentration than AOB in cluster 6a (Table 2).

### Oxidative stress

Strain PY1, as well as other AOB, have superoxide dismutase, catalase, rubrerythrin (*rdx*) and alkyl hydroperoxide reductase (*alpC*) genes (Figure 4, Table S3) but lack the gene encoding transaldolase (*tal*), which likely detoxifies ROS and functions in a non‐oxidative reaction of the pentose phosphate pathway (Table S3). Ammonia oxidation by *Nitrosopumilus* spp. DDS1, whose genome lacks sequences encoding catalase, was stimulated by adding α‐ketoglutarate to remove H_2_O_2_ from the inorganic medium (Kim et al., 2016). Comparative proteomics has revealed that AOB in the exponential growth phase express more *rdx* and *alpC* than the catalase gene to defend against oxidative stress (Zorz et al., 2018). Even if strain PY1 has multiple genes to defend against oxidative stress, we added catalase to the inorganic medium to enhance its growth. Therefore, strain PY1 is the first AOB that requires a ROS scavenger to enhance its ammonia‐oxidising activity under normal growth conditions. Considering that the expression of some genes that defend against oxidative stress depends on the growth phase and/or culture conditions (Zorz et al., 2018), the catalase gene and other ROS genes of strain PY1 might be inactive under normal growth conditions. Previous physiological and transcriptomic analyses of co‐culture experiments using AOB and NOB suggested that *Nitrobacter winogradkyi* helps reduce oxidative stress and improves the growth of *Nitrosomonas* sp. Is79 (Sedlacek et al., 2016). Similarly, physiological and proteomic approaches have demonstrated that co‐culture with marine AOA and heterotrophs decreases hydroperoxide in the medium and supports AOA cell growth (Bayer et al., 2019). Therefore, co‐culture of strain PY1 with other bacteria, such as NOB and/or heterotrophs, might effectively stimulate ammonia oxidation in strain PY1.

### *Environmental abundance and distribution of* Nitrosomonas *sp. PY1‐like sequences*

**FIGURE 5:**
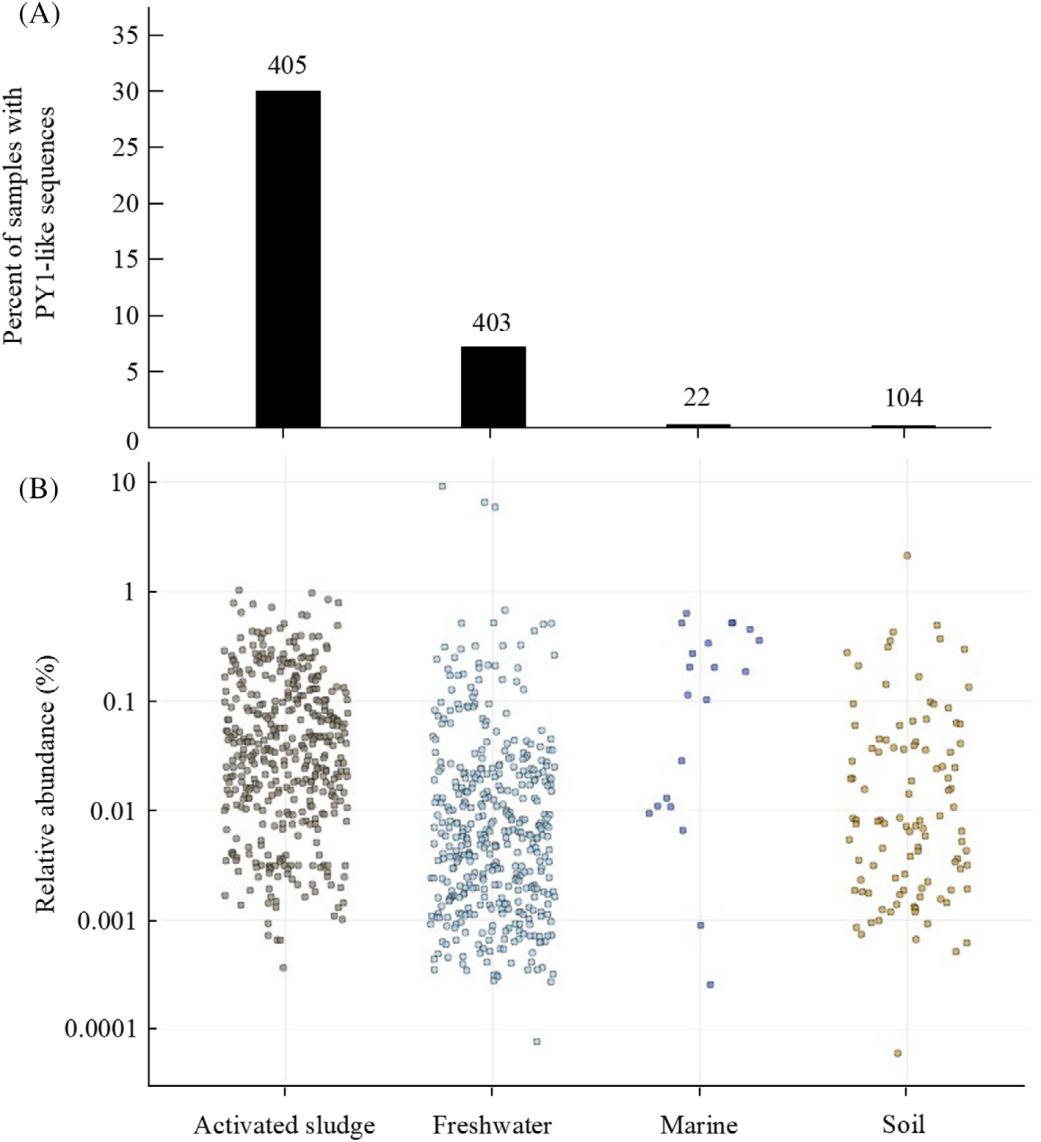
Global distribution of *Nitrosomonas* sp. PY1‐like sequences. Operational taxonomic units (OTUs) of the 16S rRNA gene were obtained from amplicon studies deposited as sequence read archive (SRA) runs. All runs with OTUs >97% identity to cultured *Nitrosomonas* sp. PY1 16S rRNA gene sequences were selected using an integrated microbial NGS platform (IMNGS). (A) The percent of SRA runs with *Nitrosomonas* sp. PY1‐like OTUs within respective environments are shown with the number of SRA runs with *Nitrosomonas* sp. PY1‐like OTUs displayed above each bar. (B) The relative abundance of all *Nitrosomonas* sp. PY1‐like OTUs are plotted in each environment.

*Nitrosomonas* sp. PY1‐like sequences were present in 934 different publicly available sequence read archive (SRA) samples deposited in GenBank (from 259,144 total SRA runs analysed with IMNGS) (Figure 5). The number of SRA samples, including representative AOB, is shown in Table S4. The ratios of the amplicon dataset, including *Nitrosomonas* sp. PY1‐like sequences, in the total amplicon data set were 30.0% in activated sludge and 7.28% in freshwater samples. Although the amplicon data set containing the *Nitrosomonas* sp. PY1‐like sequences were also detected in soil and marine samples, the percentage was considerably lower. The relative abundances of *Nitrosomonas* sp. PY1‐like sequences to total 16S rRNA sequences in each amplicon data set were 0.0003%–2%. In the four examples where the PY1 cluster comprised more than 2% of the total community, three freshwater samples (9.2%, 6.6% and 5.9%) contained data on chlorine‐free drinking water (Run accession numbers: ERR653163, ERR653166 and ERR653157, respectively). The other sample (2.1%) contained data on the soil in an olive orchard (ERR527336). Interestingly, high abundances of the PY1 cluster were detected in the three freshwater samples. We analysed the distribution of the other AOB clusters using IMNGS (Figure S6). In the activated sludge, the relative abundance of cluster 7 AOB sequences was 1%–10% whereas those of cluster PY1 and cluster 6a AOB sequences were less than 1%. In freshwater samples, the relative abundance of cluster 7 AOB sequences was less than 0.1%, whereas some relative abundances of PY1 cluster and cluster 6a AOB sequences were greater than 0.1%. Based on the comparative analysis using IMNGS, the PY1 cluster might be the predominant AOB in oligotrophic freshwater, although a distinct niche of differentiation between the PY1 cluster and cluster 6a AOB was not found.

### Challenges in isolating previously uncultured AOB and insights into their growth mechanism

AOB often have long generation times and are sensitive to environmental factors (Stein, 2019). In our previous study, we isolated three *Nitrosomonas* strains by segregating clonal microcolonies or *Nitrosomonas* cells from microbial consortia in activated sludge (Abe et al., 2017). However, it was difficult to maintain pure cultures of these cells. In this study, the addition of catalase, rather than any other organic compound, was a more effective way to increase the PY1 biomass. Considering that pyruvate and α‐ketoglutarate did not affect the ammonia‐oxidising activity of PY1 (Table S1), the supply of some organic compounds could have no specific effect on AOB strains. Previous studies have revealed that the removal of hydroperoxide by the addition of catalase (Kim et al., 2016) or in the presence or absence of associated heterotrophic contaminants (Bayer et al., 2019) enhances the ammonia oxidation activity of AOA. Based on these findings, removing hydroperoxide could effectively improve the isolation of nitrifiers and the production of sufficient biomass in pure culture. However, the effect of oxidative stress on nitrifier cells remains unclear. The molecular mechanisms underlying resistance to oxidative stress can be elucidated using genetic recombination techniques. Except for some nitrifiers (Bock et al., 1983; Klein et al., 2022; Koops et al., 1991; Sorokin et al., 2012), obtaining pure colonies of nitrifiers and/or recombinant nitrifier strains on solid media remains challenging. Medium composition and cultivation protocols that reduce oxidative stress might contribute to the isolation of novel nitrifiers and provide insights into growth mechanisms.

## CONCLUSION

We report the isolation, morphology, physiology, kinetics and genome of strain PY1, which belongs to the unclassified cluster 1 of *Nitrosomonas* and was isolated from activated sludge present in a wastewater treatment plant. Ammonia oxidation by PY1 was enhanced by the addition of catalase to an inorganic medium containing ammonia. Compared with the known *Nitrosomonas* strains, strain PY1 had a longer generation time, higher yield and required more ROS scavengers. Although the morphology and genome of strain PY1 were similar to those of the *Nitrosomonas* genus, phylogenetic analysis, including genomic information, revealed that this strain belongs to a novel clade of the *Nitrosomonas* genus. These findings broaden our understanding of ammonia‐oxidising *Nitrosomonas*, an ecologically important group in the biogeochemical nitrogen cycle and artificial nitrogen removal processes.

## AUTHOR CONTRIBUTIONS

Shuta Kikuchi, Hirotsugu Fujitani and Satoshi Tsuneda designed the study and wrote the manuscript with the help of all the authors. Shuta Kikuchi obtained sufficient biomass from the pure culture and performed the physiological experiments with the assistance of Kento Ishii and Rino Isshiki. Yuji Sekiguchi performed the genome sequencing and assembly. Shuta Kikuchi analysed the genome data along with Kento Ishii and Yuji Sekiguchi. All authors contributed to the manuscript and approved the submitted version.

## CONFLICT OF INTEREST STATEMENT

The authors declare that there are no conflicts of interest.

## Supporting information


**Figure S1.** Effect of catalase on ammonia oxidation by strain PY1. Catalase was added at a final concentration of 50 U mL^−1^.
**Figure S2**. The log‐transformed cell number in the exponential growth phase. The whole growth curve includes the lag and stationary phase. The experiments were performed in biological triplicates. Error bars indicate the standard deviation. This figure is produced from Figure 1C.
**Figure** S3. Kinetic parameters of strain PY1. Circle plots represent total ammonium uptake. The best‐fit curve was described according to Michalis–Menten equation to obtain *K*_m (app)_ and *V*_*max*_. The experiments were conducted with biological triplicates. One representative data is shown in Figure 1D.
**Figure** S4. Phylogenetic tree of the genus *Nitrosomonas* based on 16S rRNA gene sequence. The tree was constructed using the maximum likelihood algorithm with the Tamura‐Nei model in MEGA in ClustalW in MEGA 7 software. Values (%) at the branch nodes were iterated based on 1000 times bootstrapping. The scale bar corresponds to 2% estimated sequence divergence. Accession numbers are shown to the right of the microorganism names/descriptions. The sequence of strain PY1 was obtained from whole genome sequences analysed in this study (BQXC00000000).
**Figure** S5. Urea utilisation of strain PY1. The experiments were conducted using different initial cell densities and no culture replicate. Initial cell densities are (A) 10^4^ cells mL^−1^, (B) 10^5^ cells mL^−1^. Circle shows ammonium produced in the presence of catalase. Triangle shows urea produced in the presence of catalase. Square shows ammonium without catalase. Diamond shows urea without catalase.
**Figure** S6. The relative abundance of the representative AOB operational taxonomic units (OTUs) are plotted in each environment. OTUs of the 16S rRNA gene were obtained from amplicon studies deposited as sequence read archive (SRA) runs. All runs with OTUs >97% identity to cultured *Nitrosomonas* spp. 16S rRNA gene sequences were selected using an integrated microbial NGS platform (IMNGS).

**Table S1.** Effect of compounds on ammonia‐oxidising activity of strain PY1.

**Table S2** Genome overview of Nitrosomonas sp. PY1.

**Table S3.** Nitrosomonas sp. PY1 proteins with predicted functions in key metabolic pathways.

**Table S4.** Number of SRA samples in AOB.

**Table S5\** Characteristics of primer sets targeting 16S rRNA genes used for qPCR.

## Data Availability

The NCBI BioProject number for genome sequencing of Nitrosomonas sp. strain PY1 is PRJDB5489 (https://www.ncbi.nlm.nih.gov/bioproject/?term=txid1803906). Illumina raw reads were deposited in the DDBJ SRA under accession number DRA005481 (https://www.ncbi.nlm.nih.gov/sra/?term=DRA005481). The reconstructed genome sequence of strain PY1 was deposited in NCBI under the accession numbers BQXC01000001 (https://www.ncbi.nlm.nih.gov/nuccore/BQXC01000001), BQXC01000002 (https://www.ncbi.nlm.nih.gov/nuccore/BQXC01000002), BQXC01000003 (https://www.ncbi.nlm.nih.gov/nuccore/BQXC01000003), and BQXC01000004 (https://www.ncbi.nlm.nih.gov/nuccore/BQXC01000004).
