# Supplementary material for: Characterisation of bacteria representing a novel Nitrosomonas clade: Physiology, genomics and distribution of missing ammonia oxidizer
Source: Environ Microbiol Rep. 2023 Apr 20;15(5):404–16. doi: 10.1111/1758-2229.13158 (PMC10472526; doi:10.1111/1758-2229.13158)
Supplement: Supplementary file 1 — Figure S1. Effect of catalase on ammonia oxidation by strain PY1. Catalase was added at a final concentration of 50 U mL−1. Figure S2. The log‐transformed cell number in the exponential growth phase. The whole growth curve includes the lag and stationary phase. The experiments were performed in biological triplicates. Error bars indicate the standard deviation. This figure is produced from Figure 1C. Figure S3. Kinetic parameters of strain PY1. Circle plots represent total ammonium uptake. The best‐fit curve was described according to Michalis–Menten equation to obtain K m (app) and V max . The experiments were conducted with biological triplicates. One representative data is shown in Figure 1D. Figure S4. Phylogenetic tree of the genus Nitrosomonas based on 16S rRNA gene sequence. The tree was constructed using the maximum likelihood algorithm with the Tamura‐Nei model in MEGA in ClustalW in MEGA 7 software. Values (%) at the branch nodes were iterated based on 1000 times bootstrapping. The scale bar corresponds to 2% estimated sequence divergence. Accession numbers are shown to the right of the microorganism names/descriptions. The sequence of strain PY1 was obtained from whole genome sequences analysed in this study (BQXC00000000). Figure S5. Urea utilisation of strain PY1. The experiments were conducted using different initial cell densities and no culture replicate. Initial cell densities are (A) 104 cells mL−1, (B) 105 cells mL−1. Circle shows ammonium produced in the presence of catalase. Triangle shows urea produced in the presence of catalase. Square shows ammonium without catalase. Diamond shows urea without catalase. Figure S6. The relative abundance of the representative AOB operational taxonomic units (OTUs) are plotted in each environment. OTUs of the 16S rRNA gene were obtained from amplicon studies deposited as sequence read archive (SRA) runs. All runs with OTUs >97% identity to cultured Nitrosomonas spp. 16S rRNA gene sequences were selected using [file EMI4-15-404-s006.pdf]

Supporting Information

Experimental procedures

Culture conditions

PY1 was cultured in an inorganic medium containing 0.71 mM NH_4Cl. The inorganic medium was prepared by dissolving in one liter of water: NaCl (0.116 g), $MgSO_4{\cdot}7H_2O$ (0.4 g), $CaCl_2{\cdot}2H_2O$ (0.073 g), KCl (0.038 g), KH_2PO_4 (0.034 g), and trace elements (1 mL). Trace elements per liter of water were $FeCl_2$ (2.0 g), EDTA (4.3 g), $MnCl_2{\cdot}4H_2O$ (0.1 g), $CoCl_2{\cdot}6H_2O$ (0.024 g), $NiCl_2{\cdot}6H_2O$ (0.024 g), $CuCl_2{\cdot}2H_2O$ (0.017 g), $ZnCl_2$ (0.068 g), $Na_2WO_4{\cdot}2H_2O$ (0.033 g), Na_2MoO_4 (0.024 g), and H_3BO_3 (0.062 g). The initial pH was adjusted to 7.5–8.2 using HCl and $NaHCO_3$, after autoclaving. Catalase (Fuji Film Wako Pure Chemical Industries, Ltd., Osaka, Japan) was added at a final concentration of 50 or 400 U mL^{-1}. The culture was incubated in the dark without shaking unless otherwise noted.

Effects of compounds on strain PY1

PY1 was cultured for 18–41 days in the inorganic medium containing the following compounds: glucose (0.5 mM, 10 mM), fructose (0.5 mM, 10 mM), sucrose (0.5 mM), pyruvate (0.5 mM), a-ketoglutarate (0.5 mM), formate (0.5 mM, 10 mM), acetate (0.5 mM, 10 mM), citrate (0.5 mM), lactate (0.5 mM), succinate (0.5 mM, 1mM), malate (0.5 mM, 1 mM), L-alanin (1 g L^{-1}), L-cystein (1 g L^{-1}), complex nutrient: LB medium (10%), R2A medium (10%), urea (0.5 mM, 1.5 mM), catalase (50 mL^{-1}, 400 mL^{-1}). The cultures were statically incubated at 28°C in the dark. The activity and growth of PY1 were examined periodically by chemical analysis and microscopic observation.

Chemical analyses

The concentrations of ammonium (NH_4^+) and total ammonia nitrogen (NH_3 + NH_4^+) were measured using the indophenol method (Kandeler and Gerber, 1988) and Nessler's reagent (Fuji Film Wako Pure Chemical Industries, Ltd.), respectively. The nitrite concentration was determined using Griess reagent (Shinn, 1941). The absorbance was measured at wavelengths of 630 nm (for the indophenol method), 420 nm (Nessler’s reagent), and 550 nm (Griess reagent) using a Powerscan HT multiplate reader (DS Pharma Biomedical, Osaka, Japan).

Physiological experiments

To increase cell density and conduct physiological experiments, cells from stock cultures were collected by filtration (pore size 0.2 µm) and resuspended in an inorganic medium. The prepared cultures were incubated at 4, 16, 23, 28, 37, and 46°C for 32 days to determine the optimal temperature. To investigate ammonia tolerance, the cultures were incubated in an inorganic medium containing 0.71 to 7.1 mM NH_4Cl at 23°C for 32 days. To determine the generation time, specific growth rate, and growth yield, cultures were incubated in inorganic medium containing 0.56 mM NH_4Cl at 28°C and pH 8.2–8.3. The suspensions were filtered and immediately frozen at −20°C to measure ammonium and nitrite concentrations daily and were sampled to evaluate the 16S rRNA gene copy number using qPCR every alternate day. Specific growth rate (μ) was calculated from the exponential growth phase of PY1. The maximum specific growth rate (μ_{max}) was calculated using the following formula: $\mu_{max} = \mathrm{Ln}\ (X_6 / X_4) / 2$, where X_4 and X_6 are the cell densities on days four and six, respectively. The generation time (*g.t.*) was calculated using the equation $g.t. = \mathrm{Ln}\ (2) / \mu$. The growth yield was calculated from the cell density and nitrite accumulation between days 0 and 12. All nitrites were assumed to be derived from ammonia oxidation.

To calculate kinetic parameters, cell suspensions were transferred to a 5 mL glass chamber containing an inorganic medium with 0.36 mM NH_4Cl at a final volume of 3 mL. The pH was set at 8.0. The glass chambers were shaken at 23°C until no total ammonia nitrogen was detected using the Nessler reagent. During incubation, 55 µL of the samples was removed every 5–30 min, and the cells were immediately inactivated by heat shock at 95°C for 5 min. The apparent half-saturation constant ($K_{m\,(app)}$ in µM $NH_3 + NH_4^+$) for total ammonia nitrogen and the maximum total ammonia nitrogen uptake rate (V_{max} in µmol N (mg protein) $^{-1}$ h^{-1}) was calculated by fitting the Michaelis-Menten equation, as previously described (Thandar et al., 2016). As the experiment was conducted using cells instead of purified enzymes, we defined the calculated value as $K_{m\,(app)}$. NaOH was added to the samples at a final concentration of 0.15 mM for protein extraction. Cell lysates were incubated at 90°C for 30 min (Nowka et al., 2015). Protein concentrations were measured using the BCA Protein Assay Kit (TaKaRa Bio, Shiga, Japan). The $K_{m\,(app)}$ (µM NH_3) value was calculated based on culture temperature and pH according to a previously described formula (Anthonisen et al., 1976).

Urea utilization

To examine urea utilization by strain PY1, cells from stock cultures were collected using filtration (pore size 0.2 µm) and resuspended in an inorganic medium. Ammonium (0.71 mM), catalase (50 mL^{-1}), and urea (0.5 mM) were added to two different initial cell densities (10^4 or 10^5 cells mL^{-1}). The ammonium and nitrite concentrations were measured using the indophenol method and Griess reagent, respectively.

TEM and SEM

Morphological characterization of PY1 was conducted using TEM and SEM. Cells were observed based on a previously described protocol (Fujitani et al., 2014) with technical support from the Hanaichi Ultrastructure Research Institute (Okazaki, Japan).

DNA extraction

The genomic DNA of PY1 cells was extracted according using the NucleoSpin® Tissue kit (TaKaRa Bio).

Genome sequences, assembly, annotation, and analyses

Genome sequences and their reconstructions were conducted as previously described (Ushiki et al., 2018). Briefly, sequencing was performed as pair-end (300–1,000 bp inserts, Nextera XT indexed) and Nextera mate-pair (1–14 kbp insert) runs on an Illumina MiSeq instrument using V2 chemistry (2-bp × 250 bp reads). SeqPrep was used to remove adapters and merge raw reads using default settings. Reads that were not merged were trimmed and filtered using Nesoni v0.112. Genome assembly was performed using SPAdes version 2.5.0 (Bankevich et al., 2012), followed by manual curation (Sekiguchi et al., 2015). The reconstructed draft genome of strain PY1 was integrated into the DDBJ Fast Annotation and Submission Tool (DFAST) using default settings to predict and annotate the coding sequences (CDS). The relative sequences of DNA and amino acids were searched using BLAST in the National Center for Biotechnology Information (NCBI) database. The genomes of the *Nitrosomonas* sp. Is79A3 (CP002876), *Nitrosomonas* sp. AL212 (CP002552–CP002554), *N. ureae* (CP013341), *N. cryotolerans* (FSRO00000000), *N. communis* (CP011451), *N. mobilis* (FMWO01000001–FMWO010000112), *N. eutropha* (CP000450–CP000452), *N. europaea* (AL954747), and *Nitrosospira multiformis* (CP000103–CP000106) were obtained from the NCBI database. Average Nucleotide Identity (ANI) and

Average Amino acid identity (AAI) values between strain PY1 and other AOB were calculated using ANI and AAI calculators with default settings (Rodriguez-R and Konstantinidis, 2016).

phylogenetic analysis

Near full-length sequences closely related to the 16S rRNA gene of strain PY1 were collected and aligned using ClustalW in the MEGA 7 software using default settings (Kumar et al., 2016). A phylogenetic tree based on the 16S rRNA gene sequences was constructed using a maximum likelihood algorithm with the Tamura-Nei model in MEGA (Fig. S5).

Determination of PY1 growth rate using qPCR

TB Green™ Premix Ex TaqTM II (Tli RNaseH Plus; TaKaRa Bio) and Thermal Cycler Dice® Real-Time System (TaKaRa Bio) were used to quantify the 16S rRNA gene copy number of the strain PY1. PCR products amplified using NitBf and AMOr primers were purified using the Wizard SV Gel and PCR Clean-up System (Promega, Madison, WI, USA). The DNA concentration and quality were measured using a Qubit fluorometer (Thermo Fisher Scientific, Waltham, MA, USA). The purified amplicon, at a known concentration, was diluted from 10^1 to 10^9 copies/reaction to obtain a standard curve. The following thermal conditions were used for amplification: an initial denaturing step at 95°C for 30 s, followed by 40 cycles of denaturation at 95°C for 5 s, annealing and elongation at 57°C for 30 s, and melting curve analysis. The PCR efficiency was 103.9% and the R^2 value was 99.6%. As the PY1 genome harbors a single copy of the 16S rRNA gene, we regarded the copy number estimated by qPCR as the cell number.

IMNGS analysis

The full-length 16S rRNA gene sequence of PY1 was submitted to the IMNGS server (https://www.imngs.org/) (Lagkouvardos et al., 2016) to assess the distribution of phylogenetically related AOB. Sequences of at least 200 bp with a minimum identity of 97% with the 16S rRNA gene of strain PY1 were collected from the NCBI SRA.

Data availability statement

The NCBI BioProject number for genome sequencing of *Nitrosomonas* sp. strain PY1 is PRJDB5489 (https://www.ncbi.nlm.nih.gov/bioproject/?term=txid1803906). Illumina raw reads were deposited in the DDBJ SRA under accession number DRA005481 (https://www.ncbi.nlm.nih.gov/sra/?term=DRA005481). The reconstructed genome sequence of strain PY1 was deposited in NCBI under accession numbers BQXC01000001 (https://www.ncbi.nlm.nih.gov/nuccore/BQXC01000001), BQXC01000002 (https://www.ncbi.nlm.nih.gov/nuccore/BQXC01000002), BQXC01000003 (https://www.ncbi.nlm.nih.gov/nuccore/BQXC01000003), and BQXC01000004 (https://www.ncbi.nlm.nih.gov/nuccore/BQXC01000004).

References

Anthonisen, A.C., Loehr, R.C., Prakasam, T.B.S., and Srinath, E.G. (1976) Inhibition of nitrification by ammonia and nitrous-acid. *J Water Pollut Control Fed* **48**: 835-852.

Bankevich, A., Nurk, S., Antipov, D., Gurevich, A.A., Dvorkin, M., Kulikov, A.S., et al. (2012) SPAdes: a new genome assembly algorithm and its applications to single-cell sequencing. *J Comput Biol.* **19**: 455-477.

Fujitani, H., Ushiki, N., Tsuneda, S., and Aoi, Y. (2014) Isolation of sublineage I *Nitrospira* by a novel cultivation strategy. *Environ Microbiol* **16**: 3030-3040.

Kandeler, E., and Gerber, H. (1988) Short-term assay of soil urease activity using colorimetric determination of ammonium. *Biol Fertil Soils* **6**: 68-72.

Kumar, S., Stecher, G., and Tamura, K. (2016) MEGA7: Molecular Evolutionary Genetics Analysis Version 7.0 for Bigger Datasets. *Mol Biol Evol* **33**: 1870-1874.

Lagkouvardos, I., Joseph, D., Kapfhammer, M., Giritli, S., Horn, M., Haller, D., and Clavel, T. (2016) IMNGS: A comprehensive open resource of processed 16S rRNA microbial profiles for ecology and diversity studies. *Sci Rep* **6**: 33721.

McCaig, A., Embley, T., and Prosser, J. (1994) Molecular analysis of enrichment cultures of marine ammonia oxidisers. *Fems Microbiology Letters* **120**: 363-367.

Nowka, B., Daims, H., Spieck, E. (2015) Comparison of Oxidation Kinetics of Nitrite-Oxidizing Bacteria: Nitrite Availability as a Key Factor in Niche Differentiation. *Appl Environ Microbiol* **81**: 745-753.

Rodriguez-R, L.M., Konstantinidis, K.T. (2016) The enveomics collection: a toolbox for specialized analyses of microbial genomes and metagenomes. *PeerJ* **4**: e1900v1.

Sekiguchi, Y., Ohashi, A., Parks, D. H., Yamauchi, T., Tyson, G. W., Hugenholtz, P. (2015) First genomic insights into members of a candidate bacterial phylum responsible for wastewater bulking. *PeerJ* **3**: e740.

Shinn, M. (1941) Colorimetric method for determination of nitrite. *Ind. Eng. Chem.* **13**: 33-35.

Thandar, S.M., Ushiki, N., Fujitani, H., Sekiguchi, Y., and Tsuneda, S. (2016) Ecophysiology and Comparative Genomics of *Nitrosomonas mobilis* Ms1 Isolated from Autotrophic Nitrifying Granules of Wastewater Treatment Bioreactor. *Front Microbiol* **7**: 1869.

Ushiki, N., Fujitani, H., Shimada, Y., Morohoshi, T., Sekiguchi, Y., and Tsuneda, S. (2018) Genomic Analysis of Two Phylogenetically Distinct Nitrospira Species Reveals Their Genomic Plasticity and Functional Diversity. *Front Microbiol* **8**: 2637.

Voytek, M., and Ward, B. (1995) Detection of ammonium-oxidizing bacteria of the beta-subclass of the class Proteobacteria in aquatic samples with the PCR. *Applied and Environmental Microbiology* **61**: 1444-1450.

Supporting Figures

Figure legends

Fig. S1

Effect of catalase on ammonia oxidation by strain PY1. Catalase was added at a final concentration of 50 U mL^{-1}.

Fig. S2

The log-transformed cell number in the exponential growth phase. The whole growth curve includes the lag and stationary phase. The experiments were performed in biological triplicates. Error bars indicate the standard deviation. Figure S2 is produced from Figure 1C.

Fig. S3

Kinetic parameters of strain PY1. Circle plots represent total ammonium uptake. The best-fit curve was described according to Michalis-Menten equation to obtain $K_{m\ (app)}$ and V_{max}. The experiments were conducted with biological triplicates. One representative data is shown in Figure 1D.

Fig. S4

Phylogenetic tree of the genus *Nitrosomonas* based on 16S rRNA gene sequence. The tree was constructed using the maximum likelihood algorithm with the Tamura-Nei model in MEGA in ClustalW in MEGA 7 software. Values (%) at the branch nodes were iterated based on 1,000 times bootstrapping. The scale bar corresponds to 2% estimated sequence divergence. Accession numbers are shown to the right of the microorganism names/descriptions. The sequence of strain PY1 was obtained from whole genome sequences analyzed in this study (BQXC00000000).

Fig. S5

Urea utilization of strain PY1. The experiments were conducted using different initial cell densities and no culture replicate. Initial cell densities are (A) 10^4 cells mL^{-1}, (B) 10^5 cells mL^{-1}. Circle shows ammonium produced in the presence of catalase. Triangle shows urea produced in the presence of catalase. Square shows ammonium without catalase. Diamond shows urea without catalase.

Fig. S6

The relative abundance of the representative AOB operational taxonomic units (OTUs) are plotted in each environment. OTUs of the 16S rRNA gene were obtained from amplicon studies deposited as sequence read archive (SRA) runs. All runs with OTUs > 97% identity to cultured *Nitrosomonas* spp. 16S rRNA gene sequences were selected using an integrated microbial NGS platform (IMNGS).

Fig. S1

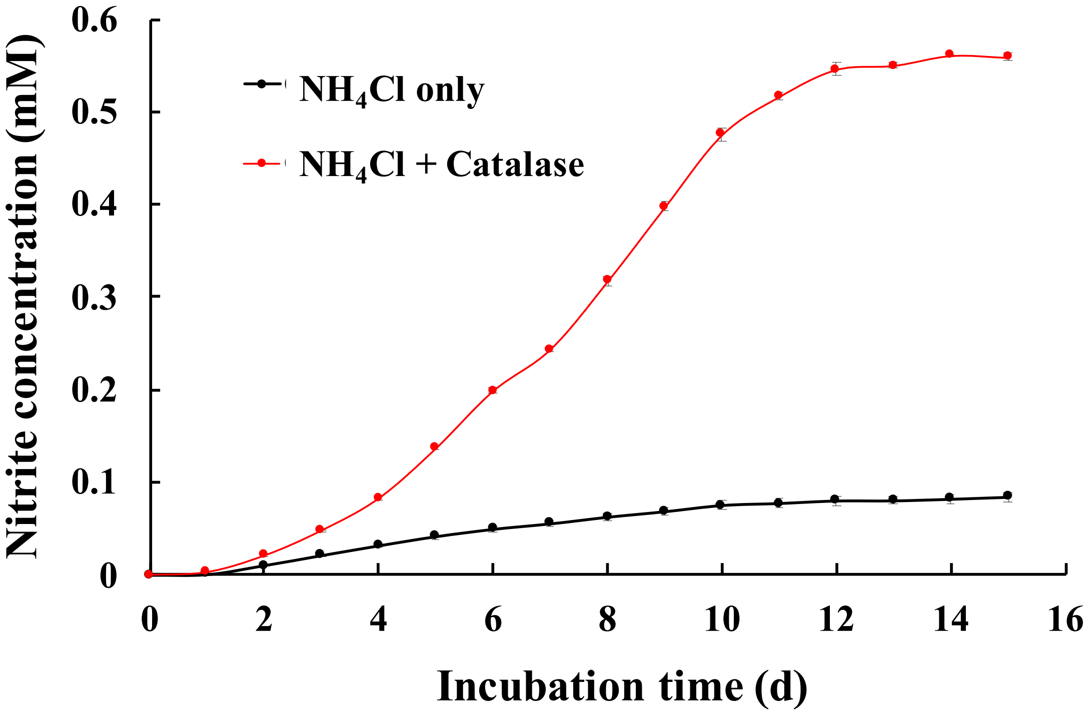
NH4Cl only
NH4Cl + Catalase
Nitrite concentration (mM)
0
0.1
0.2
0.3
0.4
0.5
0.6
0
2
4
6
8
10
12
14
16
Incubation time (d)

Fig. S2

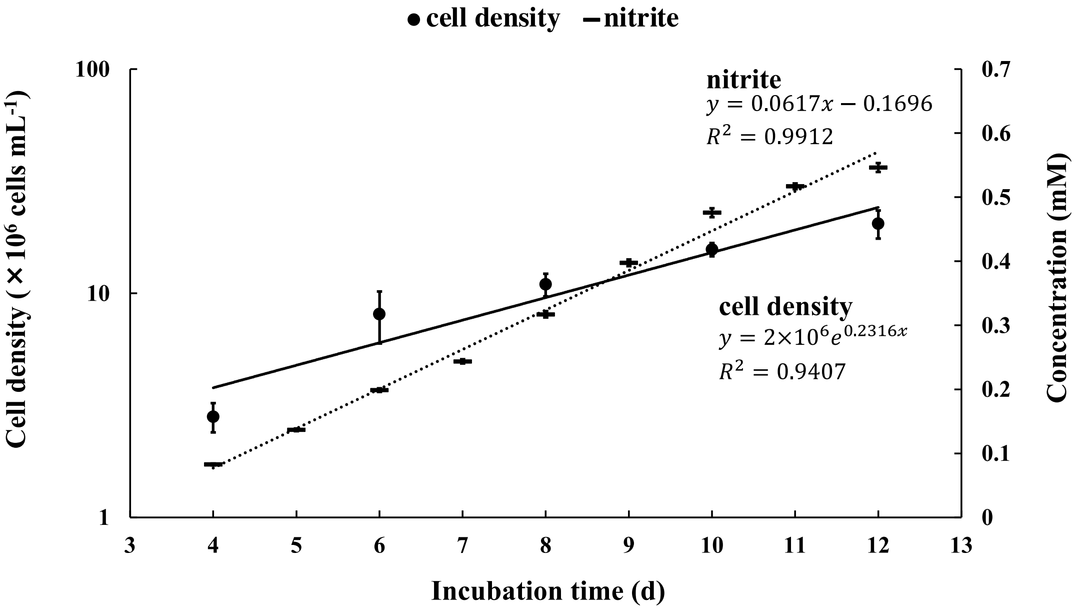

● cell density
– nitrite
nitrite
$y = 0.0617x - 0.1696$
$R^2 = 0.9912$
cell density
$y = 2{\times}10^6 e^{0.2316x}$
$R^2 = 0.9407$
Cell density ($\times 10^6$ cells mL^{-1})
Concentration (mM)
Incubation time (d)
100
10
1
0.7
0.6
0.5
0.4
0.3
0.2
0.1
0
3
4
5
6
7
8
9
10
11
12
13

Fig. S3

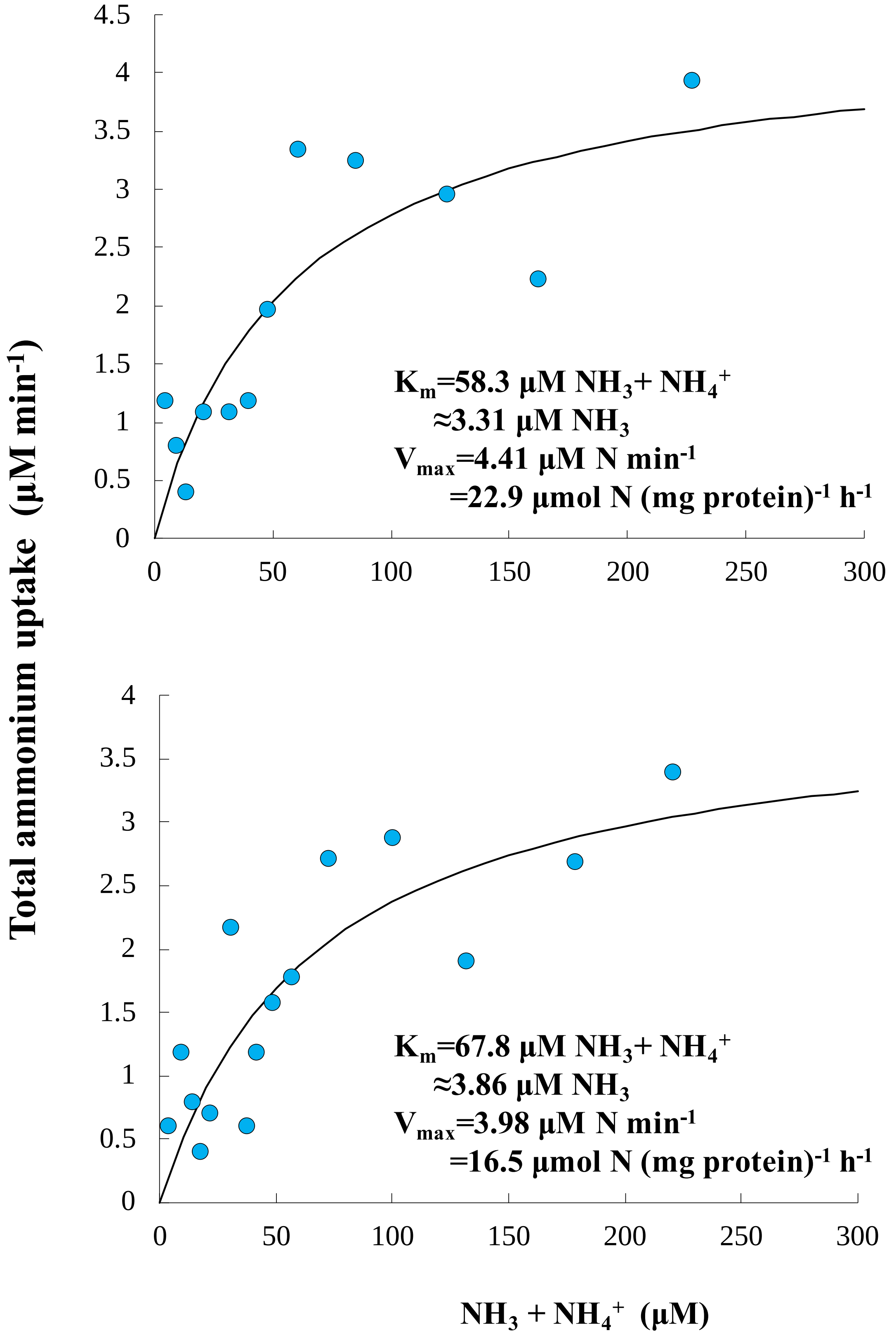
Total ammonium uptake (µM min-1)
Km=58.3 µM NH3+ NH4+
≈3.31 µM NH3
Vmax=4.41 µM N min-1
=22.9 µmol N (mg protein)-1 h-1
Km=67.8 µM NH3+ NH4+
≈3.86 µM NH3
Vmax=3.98 µM N min-1
=16.5 µmol N (mg protein)-1 h-1
NH3 + NH4+ (µM)
0
0.5
1
1.5
2
2.5
3
3.5
4
4.5
0
50
100
150
200
250
300

Fig. S4

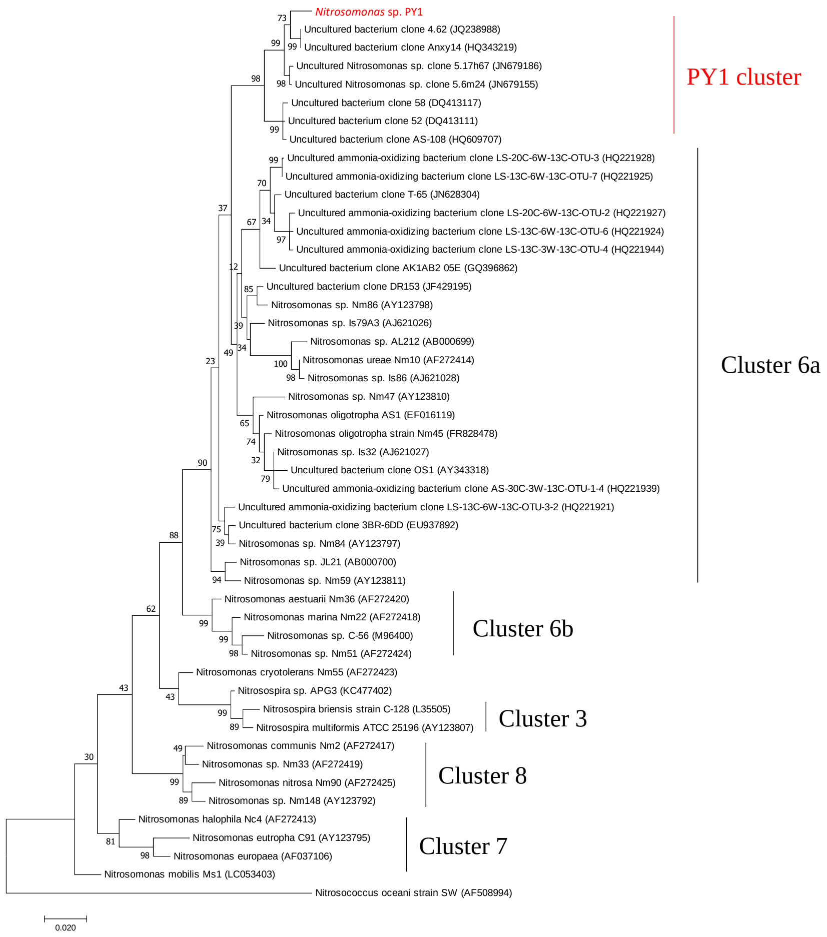

Nitrosomonas sp. PY1
Uncultured bacterium clone 4.62 (JQ238988)
Uncultured bacterium clone Anxy14 (HQ343219)
Uncultured Nitrosomonas sp. clone 5.17h67 (JN679186)
Uncultured Nitrosomonas sp. clone 5.6m24 (JN679155)
Uncultured bacterium clone 58 (DQ413117)
Uncultured bacterium clone 52 (DQ413111)
Uncultured bacterium clone AS-108 (HQ609707)
PY1 cluster
Uncultured ammonia-oxidizing bacterium clone LS-20C-6W-13C-OTU-3 (HQ221928)
Uncultured ammonia-oxidizing bacterium clone LS-13C-6W-13C-OTU-7 (HQ221925)
Uncultured bacterium clone T-65 (JN628304)
Uncultured ammonia-oxidizing bacterium clone LS-20C-6W-13C-OTU-2 (HQ221927)
Uncultured ammonia-oxidizing bacterium clone LS-13C-6W-13C-OTU-6 (HQ221924)
Uncultured ammonia-oxidizing bacterium clone LS-13C-3W-13C-OTU-4 (HQ221944)
Uncultured bacterium clone AK1AB2 05E (GQ396862)
Uncultured bacterium clone DR153 (JF429195)
Nitrosomonas sp. Nm86 (AY123798)
Nitrosomonas sp. Is79A3 (AJ621026)
Nitrosomonas sp. AL212 (AB000699)
Nitrosomonas ureae Nm10 (AF272414)
Nitrosomonas sp. Is86 (AJ621028)
Nitrosomonas sp. Nm47 (AY123810)
Nitrosomonas oligotropha AS1 (EF016119)
Nitrosomonas oligotropha strain Nm45 (FR828478)
Nitrosomonas sp. Is32 (AJ621027)
Uncultured bacterium clone OS1 (AY343318)
Uncultured ammonia-oxidizing bacterium clone AS-30C-3W-13C-OTU-1-4 (HQ221939)
Uncultured ammonia-oxidizing bacterium clone LS-13C-6W-13C-OTU-3-2 (HQ221921)
Uncultured bacterium clone 3BR-6DD (EU937892)
Nitrosomonas sp. Nm84 (AY123797)
Nitrosomonas sp. JL21 (AB000700)
Nitrosomonas sp. Nm59 (AY123811)
Cluster 6a
Nitrosomonas aestuarii Nm36 (AF272420)
Nitrosomonas marina Nm22 (AF272418)
Nitrosomonas sp. C-56 (M96400)
Nitrosomonas sp. Nm51 (AF272424)
Cluster 6b
Nitrosomonas cryotolerans Nm55 (AF272423)
Nitrosospira sp. APG3 (KC477402)
Nitrosospira briensis strain C-128 (L35505)
Nitrosospira multiformis ATCC 25196 (AY123807)
Cluster 3
Nitrosomonas communis Nm2 (AF272417)
Nitrosomonas sp. Nm33 (AF272419)
Nitrosomonas nitrosa Nm90 (AF272425)
Nitrosomonas sp. Nm148 (AY123792)
Cluster 8
Nitrosomonas halophila Nc4 (AF272413)
Nitrosomonas eutropha C91 (AY123795)
Nitrosomonas europaea (AF037106)
Cluster 7
Nitrosomonas mobilis Ms1 (LC053403)
Nitrosococcus oceani strain SW (AF508994)
0.020

Fig. S5

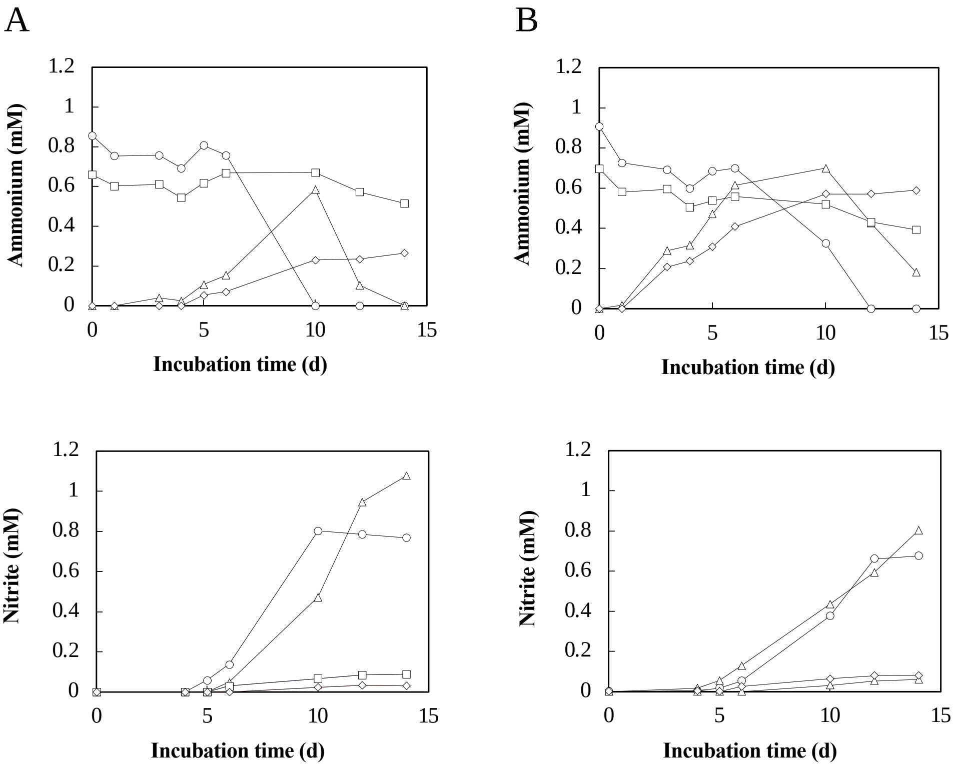
A
B
Ammonium (mM)
Nitrite (mM)
Incubation time (d)
1.2
1
0.8
0.6
0.4
0.2
0
5
10
15

Fig. S6

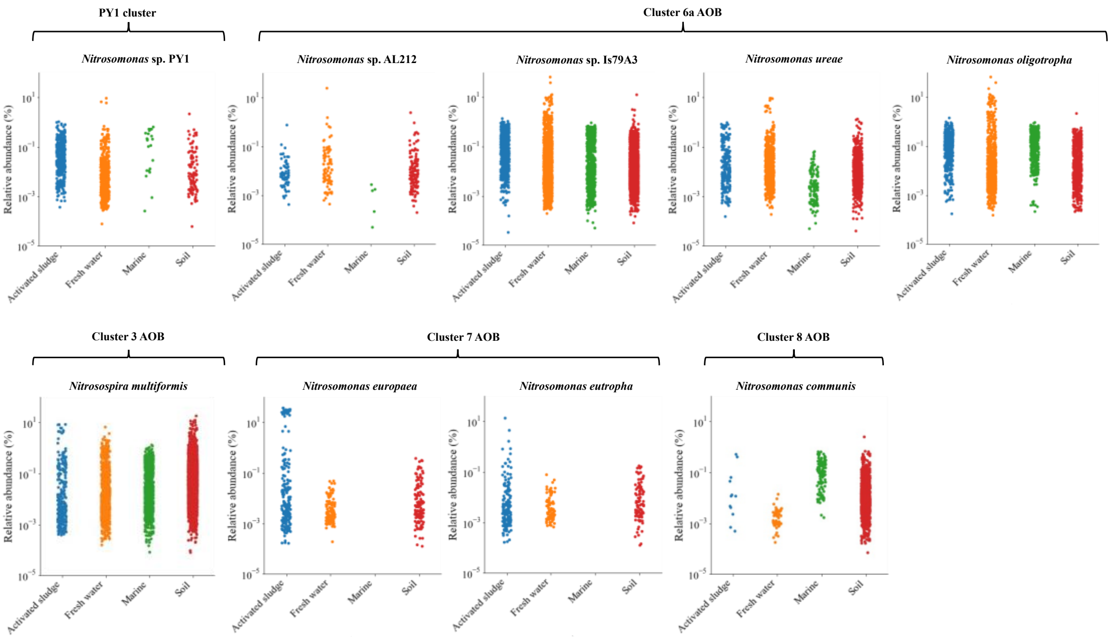
PY1 cluster
Cluster 6a AOB
Nitrosomonas sp. PY1
Nitrosomonas sp. AL212
Nitrosomonas sp. Is79A3
Nitrosomonas ureae
Nitrosomonas oligotropha
Cluster 3 AOB
Cluster 7 AOB
Cluster 8 AOB
Nitrosospira multiformis
Nitrosomonas europaea
Nitrosomonas eutropha
Nitrosomonas communis
Relative abundance (%)
Activated sludge
Fresh water
Marine
Soil
